# CTGF is overexpressed in malignant melanoma and promotes cell invasion and migration

**DOI:** 10.1038/bjc.2011.226

**Published:** 2011-06-14

**Authors:** S Braig, S Wallner, B Junglas, R Fuchshofer, A-K Bosserhoff

**Affiliations:** 1 University of Regensburg Medical School, Institute of Pathology, Franz-Josef-Strauss-Allee 11, Regensburg D-93053, Germany; 2 University of Regensburg, Institute of Anatomy, Regensburg D-93053, Germany

**Keywords:** malignant melanoma, CTGF, transcriptional regulation, invasion, hypoxia

## Abstract

**Background:**

Malignant melanoma cells are known to have altered expression of growth factors compared with normal human melanocytes. These changes most likely favour tumour growth and progression, and influence tumour environment. The induction of transforming growth factor beta1, 2 and 3 as well as BMP4 and BMP7 expression in malignant melanoma has been reported before, whereas the expression of an important modulator of these molecules, connective tissue growth factor (CTGF), has not been investigated in melanomas until now.

**Methods:**

Expression of CTGF was analysed in melanoma cell lines and tissue samples by qRT–PCR and immunohistochemistry. To determine the regulation of CTGF expression in malignant melanoma, specific siRNA was used. Additionally, migration, invasion and attachment assays were carried out.

**Results:**

We were able to demonstrate that CTGF expression is upregulated in nine melanoma cell lines and in primary and metastatic melanoma *in situ*. The transcription factor HIF-1*α* was revealed as a positive regulator for CTGF expression. Melanoma cells, in which CTGF expression is diminished, show a strong reduction of migratory and invasive properties when compared with controls. Further, treatment of normal human epidermal melanocytes with recombinant CTGF leads to an increase of migratory and invasive behaviour of these cells.

**Conclusion:**

These results suggest that CTGF promotes melanoma cell invasion and migration and, therefore, has an important role in the progression of malignant melanoma.

The Transforming Growth Factor-beta (TGF*β*) superfamily consists of over 40 members, including TGF*β*s, activins, bone morphogenetic proteins (BMPs) and nodal. They are multifunctional cytokines, which are linked to several aspects of embryonic development including the establishment of the basic embryonic body plan, differentiation morphogenesis of organs, regulation of cell proliferation, fibrosis, apoptosis, and chemotaxis (Javelaud *et al*, 2008). All members of the TGF*β* superfamily exert their cellular effects via binding to specific type I and II serine/threonine receptors. The activated type I receptor phosphorylates specific receptor-regulated Smad proteins, which then form a complex with the common partner Smad4 (Piek *et al*, 1999). Heteromeric Smad complexes efficiently translocate into the nucleus and regulate the transcription of target genes. In clear contrast to normal cells, carcinoma cells derived from several organs (for example, breast, colon and melanoma) express TGF*β* but are resistant to its growth-inhibitory effects (Jacob *et al*, 1998; Barcellos-Hoff and Akhurst, 2009; Bellam and Pasche, 2010). Therefore, it has been proposed that TGF*β* may function as a tumour promoter in advanced stages of tumour progression. In malignant melanoma, expression of the three TGF*β* isoforms positively correlates to tumour progression both *in vitro* and *in vivo* (Van Belle *et al*, 1996; Krasagakis *et al*, 1998, 1999).

Interestingly, a factor that is mainly induced by the TGF*β* isoforms, connective tissue growth factor (CTGF) (Fuchshofer *et al*, 2005, 2007; Dhar and Ray, 2010) has not been analysed in malignant melanoma yet. Connective tissue growth factor (CCN2) is a secreted and glycosylated protein built up of four specific domains, which are shared by the other members of the CCN family (Cyr61, CTGF, NOV and WISP1-3). Main functions described for CTGF are induction of migration (Igarashi *et al*, 1993), adhesion (Babic *et al*, 1999), production of extracellular matrix (Frazier *et al*, 1996; Junglas *et al*, 2009), regulation of cell cycle (Bradham *et al*, 1991), differentiation (Maeda *et al*, 2009) and wound healing (Igarashi *et al*, 1993). Because of these manifold influences on cell behaviour, CTGF and the other CCN family members became of high interest in cancer research. Until today, various correlations with cancer were shown for members of the CCN family (Dhar and Ray, 2010). Connective tissue growth factor in special was found to be overexpressed in mammary tumours, pancreatic cancer, sarcoma cancers, prostate cancers and gliomas (Yin *et al*, 2010). The purpose of this study was to determine the expression levels of CTGF in human malignant melanoma, to determine target genes and to investigate whether CTGF has a biological role in tumour development.

## Materials and methods

### Cell culture

The melanoma cell lines Mel Im, Mel Wei, Mel Ho, Mel Juso, Mel Ju, SK Mel 3 and HTZ19d were described previously (Jacob *et al*, 1998). The cell lines Mel Wei, Mel Ho and Mel Juso were derived from a primary cutaneous melanoma, Mel Im, Mel Ju, SK Mel 3, were derived from metastases of malignant melanomas. Cells were maintained in Dulbecco's modified Eagle's medium (DMEM) supplemented with penicillin (400 U ml^−1^), streptomycine (50 *μ*g ml^−1^), L-glutamine (300 *μ*g ml^−1^) and 10% fetal calf serum (FCS; Sigma, Deisenhofen, Germany) and split at a 1 : 5 ratio every 3 days. Normal human epidermal cells (NHEMs) were derived from neonatal skin. Cultivation of NHEMs was described previously (Rothhammer *et al*, 2004).

### Transfection experiments

For transient transfections 2 × 10^5^ Mel Im cells were seeded into each well of a six-well plate and transfected with 0.5 *μ*g plasmid DNA using the Lipofectamine Plus method (Invitrogen, Groningen, The Netherlands) according to the manufacturer's instructions. The antisense Sno construct was described previously (Poser *et al*, 2005). The expression vector for dominant negative HIF-1*α* was generated by introducing a stop codon (TGA) after aa 380 in the wild-type mouse HIF-1*α*-coding sequence. The resulting truncated protein thus consists of the bHLH and PAS domains, but lacks the oxygen-dependent degradation domain and all transactivation domains. Therefore, it competes with wild-type HIF-1*α* for the dimerisation partner ARNT, and may bind to HREs but does not transactivate the respective target genes. The cells were lysed 24 h after transfection, mRNA was isolated, transcribed into cDNA and qRT–PCR was performed. All transfections were repeated at least three times.

### SiRNAs and transfection procedures

HIF-1*α* siRNAs (5′-CUGAUGACCAGCAACUUGAdTdT-3′) were described before (Kuphal *et al*, 2010) and synthesised by Qiagen (Hilden, Germany). Connective tissue growth factor siRNAs (Hs_CTGF_1 and Hs_CTGF_4) were purchased from Qiagen. A siRNA (siRNA control) supplied by the company was used as control. Floating cells were transfected with the siRNAs (final concentration 50 nmol l^−1^) by the use of HighPerfect Transfection Reagent (Qiagen) in a serum-free medium according to the manufacturer's protocol. After 4 h, 10% v/v fetal calf serum was added and cells were incubated for 48 h. Thereafter the cells were lysed for mRNA isolation.

### Connective tissue growth factor protein purification

Connective tissue growth factor purification was performed as previously described (Junglas *et al*, 2009). Briefly, HEK293 cells were transfected with an expression plasmid (pDNA3.1, Invitrogen) containing human CTGF cDNA to obtain a CTGF-myc-(6x)His protein construct. Positive clones were selected by adding G418 (Invitrogen) to the medium (DMEM; 10% FBS; penicillin, 100 U ml^−1^; streptomycin, 100 *μ*g ml^−1^; all from Invitrogen) at a concentration of 250 *μ*g ml^−1^. Cells were grown in 5% CO_2_ at 37 °C. Culture medium was collected and recombinant human CTGF was purified via a three-step fast performance liquid chromatography (FPLC) system (Äktaprime, GE Healthcare, Uppsala, Sweden). For this HiTrap Heparin HP and HisTrap FF crude columns (both 5 ml; GE Healthcare) were used to take advantage of recombinant CTGF's affinity to heparin and nickel-NTA. Fractions were analysed by SDS–PAGE and silver staining. Connective tissue growth factor-containing fractions were pooled, dialysed against 0.05 M NaH_2_PO_4_, 15 mM NaCl, 0.01% (v/v) Tween 20, pH 8 and stored at −20°C until usage.

### RNA isolation and reverse transcription

Total cellular RNA was isolated from cultured cells using the RNeasy kit (Qiagen) and cDNAs were generated by reverse-transcriptase reaction performed in 20 *μ*l reaction volume containing 500 ng of total cellular RNA, 4 *μ*l of 5 × first strand buffer (Invitrogen), 2 *μ*l of 0.1 M DTT, 1 *μ*l of dN_6_-primer (10 mM), 1 *μ*l of dNTPs (10 mM) and ddH_2_O. The reaction mixture was incubated for 10 min at 70°C, 200 units of Superscript II Reverse Transcriptase (Invitrogen) were added and RNAs were transcribed for 1 h at 37°C. Reverse transcriptase was inactivated at 70°C for 10 min and the RNA was degraded by digestion with 1 *μ*l RNase A (10 mg ml^−1^) at 37 °C for 20 min.

### Analysis of gene expression by quantitative PCR

Quantitative real time-PCR was performed on a Lightcycler (Roche, Mannheim, Germany). Complementary DNA template (1 *μ*l), 0.5 *μ*l (20 mM) of forward and reverse primers and 10 *μ*l of SYBR Premix Ex Taq (TaKaRa, Shiga, Japan) in a total of 20 *μ*l were applied to the following PCR programme: 30 s 95°C (initial denaturation); 20 °C s^−1^ temperature transition rate up to 95°C for 15 s, 10 s annealing, 20 s 72 °C, 81 °C acquisition mode single, repeated for 40 times (amplification). Beta-actin is used for normalisation. Annealing and melting temperatures were optimised for each primer set (Table 1). The PCR reaction was evaluated by melting curve analysis and checking the PCR products on 1.8% agarose gels.

### Protein analysis *in vitro* (western blotting)

A total of 3 × 10^6^ cells were lysed in 200 *μ*l RIPA-buffer (Roche) and incubated for 15 min at 4 °C. Insoluble fragments were removed by centrifugation at 13 000 r.p.m. for 10 min and the supernatant lysate was immediately shock frozen and stored at −80°C. Cell lysate was loaded and separated on SDS–PAGE gradient gels (Invitrogen) and subsequently blotted onto a PVDF membrane (BioRad, Richmond, CA, USA). Thereafter, the membranes were blocked in 3% dry milk/TBS-Tween (0.1%) for 1 h and incubated with polyclonal goat anti-CTGF antibody (1 : 1000; Santa Cruz Biotechnology, Santa Cruz, CA, USA) overnight at 4°C. A 1 : 5000 dilution of rabbit anti-goat-Alkaline Phosphatase (Zymed Laboratories, San Francisco, CA, USA) was used as secondary antibody. Staining was performed using BCIP/NBT-kit (Sigma, Munich, Germany). To ensure equal loading co-staining for GAPDH or beta-actin was performed.

### Immunohistochemistry

Paraffin-embedded preparations of normal skin, nevi, primary and metastases of malignant melanomas were screened for CTGF protein expression by the avidin-biotin complex (ABC) method (DAKO-LSAB2-Kit, DAKO, Hamburg, Germany). The tissues were deparaffinated, rehydrated and incubated with primary polyclonal CTGF antibody (1 : 100; Santa Cruz Biotechnology) overnight at 4 °C. The secondary antibody supplied with the kit was incubated for 30 min at room temperature. Antibody binding was visualised using AEC-solution (for LSAB2-Kit). Finally, the tissues were counterstained by hemalaun.

### Attachment assays

Attachment assays were performed in 96-well plates. Melanoma cells were harvested by trypsinisation for 2 min, resuspended in DMEM without FCS at a density of 2 × 10^5^ cells per ml and placed in the well. Cells attached after 15 min were counted.

### Migration and invasion assay

Assays were performed using Boyden Chambers containing polycarbonate filters with 8-*μ*m pore size (Costar, Bodenheim, Germany), essentially as described previously (Rothhammer *et al*, 2005). Filters were either coated with gelatin (5 mg l^−1^) or with Matrigel (diluted 1 : 3 in H_2_O; BD Bioscience, Bedford, MA, USA). The lower compartment was filled with fibroblast-conditioned medium, used as a chemo-attractant. Melanoma cells were harvested by trypsinisation for 2 min, resuspended in DMEM without FCS at a density of 3 × 10^4^ cells per ml (migration) or 2 × 10^5^ cells per ml (invasion) and placed in the upper compartment of the chamber. After incubation at 37 °C for 4 h, the filters were collected and the cells adhering to the lower surface were fixed, stained and counted. Experiments were carried out in triplicates and were repeated three times.

### Statistical analysis

Results are expressed as mean±s.d. (range) or percent. Comparison between groups was made using the Student's unpaired *t*-test. A *P*-value <0.05 was considered statistically significant. All calculations were performed using the GraphPad Prism software (GraphPad Software Inc., San Diego, CA, USA).

## Results

Several groups including our own have reported the overexpression of TGF*β* 1, 2 and 3, in addition to BMP molecules in malignant melanoma (Rothhammer *et al*, 2005; Javelaud *et al*, 2008). Here, we were interested in screening the levels of expression of CTGF, a known modulator of TGF*β* and BMP activity and function (Kanaan *et al*, 2006).

### Analysis of CTGF expression in malignant melanoma

**Figure 1:**
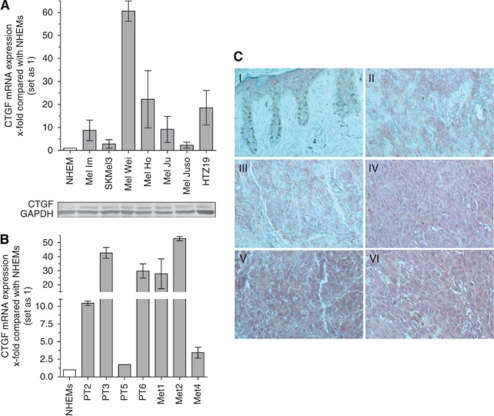
Expression of CTGF in malignant melanoma. The expression of CTGF mRNA was analysed by quantitative RT–PCR (**A**) and western blot in NHEMs and in the melanoma cell lines Mel Im, Mel Ju, Mel Juso, Mel Ho, Mel Wei, SK Mel 3 and HTZ19d. (**B**) Further, CTGF mRNA expression was determined in tissue samples of primary melanoma (PT2–PT6) and melanoma metastasis (Met1–Met4) by quantitative RT–PCR. (**C**) Connective tissue growth factor protein expression was analysed by immunohistochemistry in skin (I), primary melanoma (II and III) and metastasis of malignant melanoma (IV, V and VI). Strong expression of CTGF protein was detected in primary melanoma and metastases as shown by red AEC staining (200-fold magnification). Bars show the mean±s.d. of three independent experiments, measurements were performed in triplicates. The colour reproduction of this figure is available at the *British Journal of Cancer* online.

We first analysed the expression of CTGF in seven melanoma cell lines by using quantitative RT–PCR (qRT–PCR). Expression of CTGF was detectable in all of the analysed cell lines, whereas NHEMs did not show expression of CTGF (Figure 1A, upper part). Expression of CTGF in the melanoma cell lines was confirmed on protein level (Figure 1A, lower part). Further, in mRNA preparations of four primary melanomas and three melanoma metastases CTGF mRNA was measured by qRT–PCR. Strong expression of CTGF mRNA compared with melanocytes was observed in three out of four primary melanomas and all metastases (Figure 1B).

The *in vitro* results were extended to an *in vivo* setting by comparing the expression levels of CTGF in melanoma tissues by immunostaining sections of primary melanoma and metastasis of malignant melanoma (Figure 1C). We were able to detect an intense staining of primary melanoma and metastasis sections, thereby confirming upregulation of CTGF expression during transformation of malignant melanoma.

### HIF-1*α* functions as possible regulator of CTGF expression

**Figure 2:**
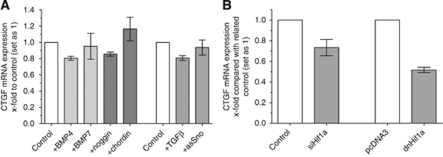
Analysis of regulation of CTGF by HIF-1*α*. (**A**) Mel Im cells were treated with recombinant BMP4 or BMP7, respectively. Quantitative RT–PCR analysis revealed no difference in CTGF mRNA expression levels compared with untreated cells. Inhibition of BMP signalling by treatment of the cells with noggin and chordin had no effect on CTGF mRNA expression. Neither treatment of melanoma cells with TGF*β*1 nor transfection of Mel Im cells with an antisense Sno construct influenced mRNA expression of CTGF. (**B**) Transient transfection of Mel Im cells with siRNA against HIF-1*α* resulted in diminished CTGF mRNA expression. Moreover, transfection of Mel Im cells with a dominant-negative HIF-1*α* construct strongly reduced CTGF gene expression compared with cells treated with pcDNA3 control vector. Bars show the mean±s.d. of three independent experiments, measurements were performed in triplicates.

It is known that members of the TGF*β* superfamily are involved in regulation of CTGF expression (Dhar and Ray, 2010). Moreover, we and others were able to determine Bone Morphogenetic Protein 4 (BMP4), BMP7 and TGF*β* as important modulators of melanoma initiation and progression (Poser *et al*, 2005; Rothhammer *et al*, 2005, 2007; Javelaud *et al*, 2008). To analyse a potential regulation of CTGF gene expression by BMPs, we incubated Mel Im melanoma cells with recombinant BMP4, BMP7 or the BMP inhibitors noggin and chordin, respectively. Quantitative RT–PCR experiments revealed no difference in CTGF mRNA expression after treatment with recombinant BMP4, BMP7, noggin and chordin (Figure 2A). Next, we investigated whether TGF*β* regulates expression of CTGF. Neither treatment of melanoma cells with TGF*β*1 nor transfection of Mel Im cells with an antisense Sno construct (Poser *et al*, 2005) influence CTGF mRNA expression (Figure 2A).

Shimo *et al* (2001) showed that CTGF expression increases in response to hypoxia in breast cancer cells. Additionally, it is known that the hypoxic induction of CTGF is directly mediated by HIF-1*α* (Higgins *et al*, 2004; Fuchshofer *et al*, 2009). On the basis of these studies we determined the effect of hypoxia on regulation of CTGF expression in melanoma. Recent studies of our group revealed constitutive strong activity of HIF-1*α* even under non-hypoxic conditions (Kuphal *et al*, 2010). Transfection of the melanoma cell line Mel Im with either the previously described siRNA against HIF-1*α* (siHIF-1*α*) or the formerly characterised dominant-negative construct of HIF-1*α* (dnHIF-1*α*) (Maeda *et al*, 2009; Kuphal *et al*, 2010) resulted in strong downregulation of CTGF mRNA expression in comparison with negative control siRNA- or vector-only transfected cells (Figure 2B).

### Regulation of BAMBI and BMP7 by CTGF

**Figure 3:**
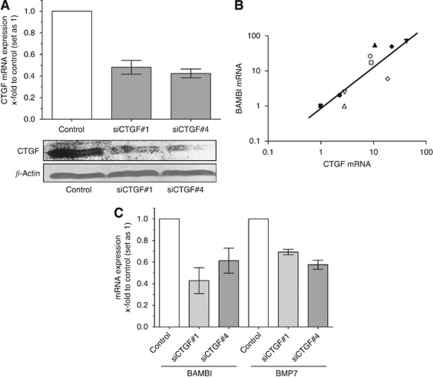
Connective tissue growth factor regulates expression of BAMBI and BMP7. (**A**) Reduction of CTGF expression was achieved by transient transfection of Mel Im cells with siRNAs against CTGF (siCTGF#1 and siCTGF#4). Transfection with scrambled siRNA served as control. Strong reduction of CTGF expression was observed after 48 h in the melanoma cell line Mel Im on mRNA and protein level. (**B**) Correlation of CTGF and BAMBI mRNA expression profiles in melanoma cell lines. Each symbol represents one melanoma cell line. (**C**) Transient transfection of melanoma Mel Im cells with both siRNAs against CTGF resulted in reduced expression of BAMBI and BMP7 mRNA levels compared with control cells, as determined by qRTPCR. Bars show the mean±s.d. of three independent experiments, measurements were performed in triplicates.

To screen for target genes of CTGF in malignant melanoma, we used a siRNA approach to downregulate CTGF expression. Transfection of Mel Im cells with specific siRNAs against CTGF (siCTGF#1 and siCTGF#4) resulted in ∼50% reduced mRNA expression of CTGF (Figure 3A, upper part). Western blot experiments confirmed diminished protein expression of CTGF after treatment with the siRNAs on protein level to 62 or 59% compared with control, respectively (Figure 3A, lower part). As BAMBI is known to be a modifier of the BMP signalling pathway, we were interested whether CTGF is involved in the regulation of BAMBI expression. Interestingly, a correlation was observed between CTGF and BAMBI expression levels in melanoma cell lines (*r*=0.854, Figure 3B). Further, downregulation of CTGF expression by siRNAs against CTGF resulted in reduced BAMBI expression (Figure 3C). Interestingly, BMP7 expression is significantly downregulated after transfection of CTGF siRNAs (Figure 3C), whereas BMP4 expression was not reduced (data not shown). As transfection of Mel Im cells with each of the CTGF siRNAs diminished the expression of CTGF, BAMBI and BMP7 to a similar extent, we performed the following assays with siCTGF#4.

### Connective tissue growth factor promotes migration and invasion of malignant melanoma cells

**Figure 4:**
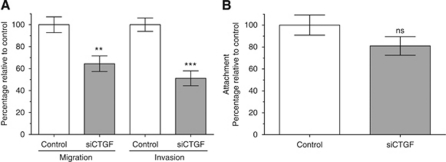
Effect of reduction of CTGF expression. (**A**) Migration and invasion assays using the Boyden Chamber model revealed a significant reduction of the migratory and invasive potential after transient downregulation of CTGF expression, whereas attachment of the cells (**B**) was not significantly changed. Bars show the mean±s.d. of three independent experiments, measurements were performed in triplicates. ^**^P<0.01; ^***^P<0.001.

Next, we analysed the functional influence of CTGF in malignant melanoma. Therefore, we transfected Mel Im cells with CTGF siRNA#4 and performed Boyden chamber assays. As shown in Figure 4A, the migratory as well as the invasive properties of the melanoma cells were clearly affected by the downregulation of CTGF via siRNA. Interestingly, attachment was only slightly regulated after siRNA transfection (Figure 4B), suggesting that other means lead to the pronounced effects on migration and invasion.

### Functional impact of CTGF treatment on melanocytes

**Figure 5:**
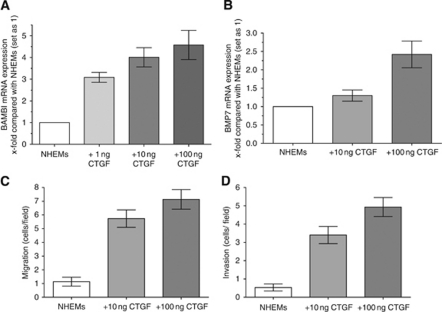
Treatment of NHEMs with CTGF results in increased expression of BAMBI and BMP7 and enhanced migratory and invasive potential. (**A** and **B**) Normal human epidermal melanocytes were treated with increasing concentrations of recombinant CTGF for 24 h. Quantitative RT–PCR revealed enhanced expression of BAMBI (**A**) and BMP7 mRNA (**B**) levels in NHEMs incubated with CTGF compared with control cells. (**C**) Connective tissue growth factor-treated cells showed strongly increased migratory potential of up to 700% when compared with untreated melanocytes. (**D**) In addition, invasive potential of NHEMs was dose dependently enhanced up to 500% after treatment with recombinant CTGF compared with control cells. Bars show the mean±s.d. of three independent experiments, measurements were performed in triplicates.

In the last set of experiments, we treated NHEMs with increasing concentrations of recombinant CTGF and the appropriate buffer control, respectively, and determined the expression of CTGF target genes. We were able to demonstrate an induction of both, BAMBI and BMP7, mRNA expression (Figures 5A and B). Finally, we analysed whether treatment with CTGF influences the migratory and invasive potential of NHEMs. We observed a strong dose-dependent increase in both migratory and invasive behaviour of the melanocytes treated with recombinant CTGF compared with untreated control cells (Figures 5C and D).

## Discussion

It is known that members of the TGF*β* superfamily have an important role in the progression of malignant melanoma. Transforming growth factor beta inhibits proliferation and DNA synthesis of normal melanocytes, whereas melanoma cells escape from these suppressive effects. Moreover, tumour cells express TGF*β* isoforms 1–3 at high levels, thereby stimulating tumour progression in an autocrine and paracrine manner (Lasfar and Cohen-Solal, 2010). Several studies described CTGF as a modulator of TGF*β* expression and activity in different cell types (Nguyen and Goldschmeding, 2008; Dhar and Ray, 2010). In addition, it was shown that TGF*β* induces CTGF expression (Fuchshofer *et al*, 2005, 2007; Cicha and Goppelt-Struebe, 2009). Until now, the functional properties of CTGF were mainly addressed to its profibrotic effect in numerous tissues (Gressner and Gressner, 2008; Chen *et al*, 2009). An upregulation of CTGF in different fibrotic disorders was mainly correlated with the overexpression of TGF*β*1 and 2 (Chen *et al*, 2009).

In cancer, expression of CTGF has been linked with both progression and inhibition of oncogenic processes, depending on sites and types of cancer (Chu *et al*, 2008). Connective tissue growth factor was found to be overexpressed in mammary tumours, pancreatic cancer, sarcoma cancers, prostate cancers (Yang *et al*, 2005) and gliomas (Yin *et al*, 2010).

Despite numerous studies showing the importance of TGF*β* signalling in malignant melanoma, the role of CTGF has not been addressed yet. Therefore, we studied expression and functional relevance of CTGF in melanoma cell lines.

In malignant melanoma CTGF mRNA overexpression was identified by *in situ* hybridisation in desmoplastic malignant melanoma, but not in amelanotic malignant melanoma (Kubo *et al*, 1998). Based on this mRNA study of Kubo *et al* we analysed CTGF expression in different melanoma cell lines compared with normal melanocytes (NHEMs). Connective tissue growth factor mRNA and protein was expressed in all melanoma cell lines, whereas no signal was observed in NHEM cells. Additionally, CTGF expression was also detected in primary melanomas tissue samples and in melanoma metastasis samples by immunohistochemistry, indicating that CTGF is involved in the progression of malignant melanoma.

As TGF*β*1, BMP4 and BMP7 are upregulated in melanoma (Rothhammer *et al*, 2005, 2007), we were interested whether CTGF expression in the melanoma cells is mediated by these cytokines. We found that the CTGF overexpression is not induced by members of the TGF*β* superfamiliy and its regulators in melanoma cell lines: Neither TGF*β*1, BMP4 nor BMP7 have a significant effect on the CTGF expression. Also treatment with modulators of the TGF*β* superfamily like noggin, chordin and transfection with an antisense Sno construct did not influence the expression of CTGF. However, knockdown of HIF-1*α* using specific siRNA or a dominant-negative expression construct, respectively, led to a strong downregulation of CTGF mRNA. In a recent study we found a constitutive HIF-1*α* activity in malignant melanoma, which could be the cause of the observed CTGF overexpression (Kuphal *et al*, 2010). The responsiveness of CTGF on activation of HIF-1*α*, which was described in previous studies, might be a link to an enhanced angiogenesis as it was found for CTGF in prostate cancer tumourigenesis (Yang *et al*, 2005).

A further involvement of CTGF in malignant melanoma formation was identified by investigating migration and invasion rates after modulating CTGF expression in melanoma cells or treatment of melanocytes with recombinant CTGF. We were able to show that knocking down CTGF expression led to a significant reduction of migration and invasion behaviour of melanoma cells. In addition, treatment of melanocytes with recombinant CTGF resulted in increased migratory and invasive potential compared with control cells, thereby further confirming the impact of CTGF on tumour progression. Hoek *et al* (2004) studied the expression profiles of melanocytes compared with invasive melanoma cells by microarray analysis. They revealed that expression of CTGF is upregulated in advanced stages of melanoma progression compared with melanocytes, which is in line with our studies. The impact of CTGF on migratory potential was also observed in other tumour types, for example, in glioblastoma multiforme cells, where an overexpression of CTGF led to an increased migratory potential (Yin *et al*, 2010).

In addition, we were able to accentuate that treatment of normal melanocytes with recombinant CTGF caused an increase in BMP7 expression, whereas knocking down CTGF expression via a specific siRNA led to diminished BMP7 expression levels in melanoma cells. BMP7 was shown to be upregulated in malignant melanoma (Rothhammer *et al*, 2007; Hsu *et al*, 2008). These data may indicate that BMP7 and CTGF function in one pathway to promote tumour progression. As CTGF expression was not stimulated by BMPs, we suggest that CTGF is an upstream regulator of BMPs in malignant melanoma. Additionally, we could show that CTGF not only increases BMP7 but also BAMBI expression, the pseudo-receptor of the TGF*β* superfamily. BAMBI (BMP- and activin membrane bound inhibitor) forms stable complexes with type II receptors, thereby inhibiting activation of Smad proteins and subsequently TGF*β* signal transduction. It is tempting to speculate that the CTGF induced increase of BAMBI expression led to an alteration of the TGF*β* signalling. Transforming growth factor beta usually has an anti-proliferative function; however, during tumourigenesis this effect is reverted (Lasfar and Cohen-Solal, 2010). Next to the previously described overexpression of the oncogenic proteins SKI and SnoN (Medrano, 2003; Poser *et al*, 2005), an increase of BAMBI expression could be an additional trigger in the functional switch of TGF*β* in malignant melanoma.

In summary, our studies show that CTGF is strongly expressed in malignant melanoma cells as well as in human tissues of primary melanoma and metastasis of malignant melanoma. Moreover, we were able to demonstrate that CTGF expression in malignant melanoma is regulated by hypoxia-inducible factor HIF-1*α*, whereas members of TGF*β* superfamily have no impact on modulation of CTGF expression levels. Connective tissue growth factor in turn controls expression of BMP7 and the TGF*β* pseudo-receptor BAMBI and contributes to tumour progression by increasing migratory and invasive potential of melanoma cells. These results suggest that CTGF has an important biological function in human malignant melanoma.

## Figures and Tables

**Table 1:** Oligonucleotide sequences

**Gene**	**Primer sequences** **(forward/reverse)**
*CTGF*	5′-CAGAACCACCACCCTGCCG-3′
	5′-CGTACATCTTCCTGTAGTACA-3′
*BAMBI*	5′-CGATGTTCTCTCTCCTCCCAG-3′
	5′-AATCAGCCCTCCAGCAATGG-3′
*BMP7*	5′-GCCAGCCTGCAAGATAGCCATTTCC-3′
	5′-GAGCACCTGATAAACGCTGATCCGG-3′
*β-Actin*	5′-CTACGTCGCCCTGGACTTCGAGC-3′
	5′-GATGGAGCCGCCGATCCACACGG-3′
